# Perovskite Thin Film Consisting with One-Dimensional Nanowires

**DOI:** 10.3390/ma11091759

**Published:** 2018-09-18

**Authors:** Xinli Li, Yongchao Chen, Lihua Li, Jinliang Huang

**Affiliations:** 1 School of Material Science and Engineering, Henan University of Science and Technology, Luoyang 471003, China; chen1yong2chao3@163.com (Y.C.); lilihua7818@163.com (L.L.); 2 Collaborative Innovation Center of Nonferrous Metals, Henan University of Science and Technology, Luoyang 471003, China

**Keywords:** perovskite thin film, one-dimensional nanowires, DMSO solvent, optical properties

## Abstract

Organic-inorganic hybrid perovskite solar cells had attracted extensive attention due to their high-power conversion efficiency and low cost. The morphology and structure of the light absorption layer are crucially important for the device performance. The one-dimensional or two dimensional nano-structure perovskite material exhibits better optical and electrical properties than three-dimensional bulk perovskite. In this article, the perovskite CH_3_NH_3_PbI_3_ thin films with one-dimensional nanowires structure were prepared while using the solution method with N,N-Dimethylformamide (DMF) and dimethyl sulfoxide (DMSO) mixed solvent under atmospheric environment. During the perovskite thin films growth, the DMSO solvent as a structure directing agent played a guiding role in the formation of nanowires. The effects of DMSO solvent added ratio on the perovskite thin film structure, morphology, optical properties, and the device performance were studied. By changing the ratio of DMSO solvent added can effectively adjust the orientation order and optical properties of the nanowires perovskite thin films. The results showed that the best ratio of DMSO solvent added in the mixed solvent was 10%. The high order orientation of the perovskite thin film with nanowires forest was obtained. It showed the high optical absorption and electrical properties. The perovskite absorption layer presents ordered and dispersed nanowires forest; the device power conversation efficiency is increased by 50% when compared with the perovskite layer presents disordered nanowires.

## 1. Introduction

In the past few years, organic-inorganic hybrid perovskite materials CH_3_NH_3_PbX_3_ (X = Cl, Br, I) have been received great attention owing to their high optical absorption coefficient, micron-sized carrier diffusion length, double charge transfer, and long carrier lifetime [1,2,3]. Perovskite materials have been introduced into solar cells as light absorption layers, and the power conversion efficiency of solar cells increased from 3.8% to 23.3% [4]. In the perovskite solar cells, the morphology and structure of the optical absorption layer played an important role in the device performance. The structure and morphology of the optical absorption layer are mainly related to the preparation method, process parameters and so on. The preparation method of the perovskite thin films can be divided into three kinds, solution method, gas-phase method, and gas-assisted liquid-phase method. Among these methods, the solution method is the commonly used because of its simplicity and operability. With the maturity of the preparation methods, some new perovskite thin films preparation methods were appeared, including water molecular engineering [5,6], solvent and anti-solvent engineering [7,8,9] additives, and ammonia gas induced and ambient annealing methods [10,11] have been developed to improve the films quality [12,13]. The solvent engineering [14,15] has been proved to be the easiest and effective way to prepare high quality film among these methods.

Currently, most of the light absorption layer of the perovskite solar cell is polycrystalline perovskite thin film with three-dimensional (3D) structure. However, the introduction of many defects and grain boundaries in 3D bulk perovskite is unavoidable, which could degrade the optoelectronic properties [16]. Based on the random walk model in nanocrystalline system with grain boundaries [17], carrier transport in 3D perovskite structure may not be rapid enough although carrier diffusion length reaches over 1 μm. So, the low-dimensional perovskite materials have been developed, such as two-dimensional (2D) and one-dimensional (1D). Ultrathin 2D perovskite flakes [18] and 2D perovskite nanosheets [16] have been produced due to the high quantum efficiency and excellent photoelectric properties. The photogenerated carriers transport and separation at the interface of hole transport layer and perovskite layer in the 1D perovskite materials were faster than that in the 3D materials [19]. The 1D perovskite nanowires structure presents faster carrier extraction ability [20]. However, the single-crystalline perovskite nanowires with well-defined structures have higher photoluminescence quantum yields, larger carrier mobilities, and longer carrier diffusion lengths [21,22,23]. 1D perovskite nanowires can be used in field emission transistor [21,24] and solar cell [25]. In the recent literature report, the order of perovskite 1D nanowires is not high. If the highly ordered and dispersed nanowires perovskite thin films can be obtained with solvent engineering, it is beneficial to the carrier transport. If the carriers transport can be accelerated, it is very important for solar cells and light emitting diode (LED) applications.

During the solvent engineering, the nucleation and crystallization rate of the perovskite film can be adjusted by adding different proportions of solvent into the precursor solutions. For example, the 1,8-diiodooctane (DIO) additive was added into the perovskite precursor solution to adjust the morphology of the perovskite film [26]. A small amount of CHP (N-cyclohexylpyrrolidone) was added into the precursor solution to form a homogeneous nucleation site in order to control the morphology of the surface of the perovskite film [27]. It has been reported that the perovskite nanowires were prepared by the dropwise addition of toluene while using a mixed solvent of isopropanol and DMF or a mixed solution of DMF and acetonitrile [28,29]. The nanowires prepared by the rapid crystallization method using a single solvent DMF have a size of several micrometers, and it is difficult to regulate the directionality and crystallinity of the nanowires. The crystallization process is slowed by the addition of solvent DMSO, which forms nanowires forest of hundreds of nano-meters by self-assembly during the crystallization process. The 1D nanowire structure thin film has a faster separation rate and better transportability at the interface than the multi-dimensional structure [19,30].

In combination with the above problems, we report a simple solution method for fabricating 1D organic-inorganic hybrid perovskite CH_3_NH_3_PbI_3_ nanowires with DMF and DMSO mixed solvents under atmospheric environment. The nanowires perovskite thin film structure, morphology and optical performance are sensitive to the ratio of DMSO solvent added. Interaction between DMSO and PbI_2_ was slowed down; the crystallization rate of PbI_2_ was slowing down, and the intermediate phase CH_3_NH_3_I-PbI_2_-DMSO was introduced into the precursor solution. With the DMSO solvent, it promoted uniform nucleation, improved the morphology and crystallinity, and produced the film of nanowires structure with high crystallinity and order orientation. The high order orientation nanowires perovskite thin film presents the preferable optical absorption and electrical properties. The effect of DMSO added ratio on the precursor solution fluorescence performance, the perovskite structure, morphology, and optical and electrical properties have been investigated. The best ratio of DMSO solvent added also has been optimized.

## 2. Materials and Methods

### 2.1. Materials

The reactants included CH_3_NH_3_I and PbI_2_. The solvent included N,N-dimethylformamide (DMF) and dimethyl sulfoxide (DMSO). The information about these reactants and DMF solvent can be found in literature [31]. The DMSO solvent was purchased from Chengdu Kelon Chemical Reagent Factory (Chengdu, China). All of the reactants and solvents were not purified again. In this experiment, 2.0 × 2.0 cm SnO_2_:F (FTO) glass was used as the substrate. The FTO substrate cleaning method can be referred to the literature [32]. Finally, the FTO substrate was placed in the container with the beaker of ethanol.

### 2.2. Perovskite Thin Films and Solar Cells Fabrication

The molar ratio of CH_3_NH_3_I and PbI_2_ is 1:1. Then, these reactant powders were added into the DMSO and DMF mixed solvents, and the total volume of solvent was 2 mL. A homogeneous perovskite precursor solution can be obtained by ultrasonic oscillation with 10 h at 70 °C. In this work, the other parameters are kept unchanged, just changing the ratio of DMSO and DMF in solvents. The volume ratios of solvent DMSO set as 0%, 5%, 10%, 15%, and 20%, respectively. The DMSO different ratios were labeled with numbers a, b, c, d, and e.

**Figure 1:**
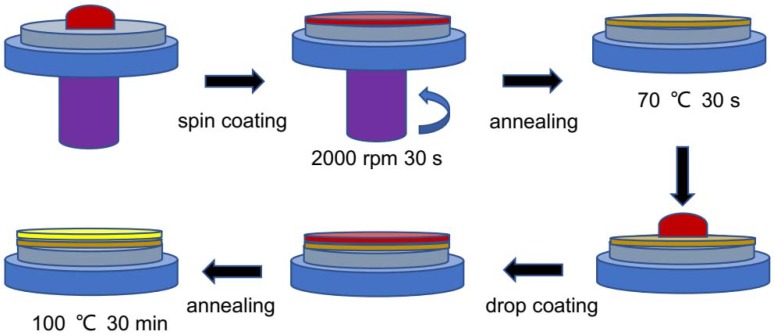
Schematic illustration of the perovskite thin films preparation progress.

**Figure 2:**
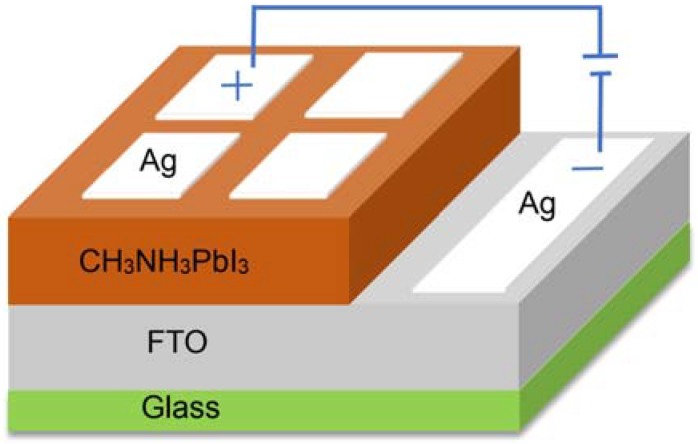
A schematic draw of the perovskite solar cell structure.

The perovskite thin films were prepared with two step spin-coating method. Firstly, perovskite precursor solution was spin-coated into the FTO substrate at 2000 rpm for 30 s in the room temperature atmospheric environment. Then put it into the drier at 70 °C for 30 s formed a seed layer. Secondly, take appropriate amount perovskite precursor solution was drop-coated on the seed layer evenly. Subsequently, the samples were annealed in a drying oven at 100 °C for 30 min to obtain the CH_3_NH_3_PbI_3_ films. Figure 1 shows the schematic diagram of perovskite thin films preparation progress. Finally, the electrode was produced by scraping a layer of conductive silver glue on the perovskite thin film. The structure of the perovskite solar cell was presented in Figure 2.

### 2.3. Material and Device Characterization

Optical properties of perovskite precursor solution were measured and characterized by F-280 Fluorescence Spectrophometer (Tianjin Gangdong Scientific and Technical Development Co., Tianjin, China). The excitation wavelength was set at 325 nm, and the measuring wavelength range was from 400 nm to 850 nm. The thermogravimetric-differential scanning calorimetry (TG-DSC) of the precursor solution was monitored by thermal analyzer of STA409PC from Germany (NETZSCH Group, Gebrüder, Germany). The structure of the perovskite films was characterized by D8 ADVANCE X-ray diffractometer (Cu target, λ = 1.54060 Å; 2θ = 10°–50°, Bruker Inc., Billerica, MA, USA). Perovskite morphology was measured by VEGA3TESCAN scanning electron microscope (TESCAN ORSAY HOLDING, Brno, Czech Republic). The Ultraviolet-visible light absorption properties were characterised by UV-2600 (Scientific Instruments, Inc., Tokyo, Japan). Transient photocurrent was tested by the electrochemical workstation of CHI660d (Shimadzu Inc., Shanghai, China), and the applied voltage is 0.5 V. The effective area of the solar cell is 0.25 cm^2^. Photocurrent density-photovoltage characteristics were tested by Keithley 2400 Source Meter (AM1.5G, 100 mW/cm^2^, Zolix ss150 Solar Simulator, Ektronix, Inc., Beaverton, OR, USA).

## 3. Results and Discussion

### 3.1. TG-DSC Analysis

**Figure 3:**
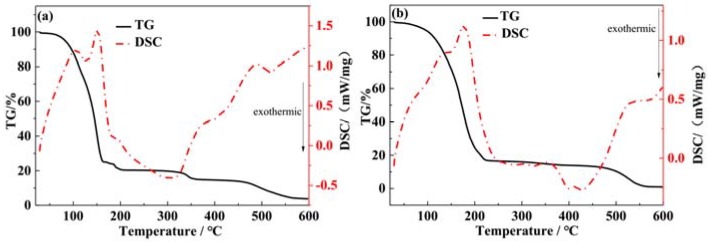
The TG-DSC curves with different ratios of dimethyl sulfoxide (DMSO) (**a**) 0%; (**b**) 10%.

Firstly, the TG-DSC curves of the perovskite precursor solution were shown in Figure 3. The temperature range was 25 °C–600 °C. To investigate the effect of DMSO solvent added on the perovskite thin film growth, we compared the differences of the TG-DSC curves before and after the DMSO solvent added. Figure 3a shows the TG-DSC curves before the DMSO solvent added, and Figure 3b shows the TG-DSC curves with a ratio of 10% DMSO solvent added. In Figure 3a, there are three significant weightlessness behaviors in the TG curve. Between 50 °C and 150 °C ranges, the TG curve shows falling rapidly, and the decrease extent is close to 80%. Due to the solvent, the DMF boiling point is 152.8 °C, so the TG curve (in Figure 3a) decreases rapidly with the DMF solvent volatilization. During this temperature range, there are two endothermic peaks in the DSC curve (see in Figure 3a). After the DMSO solvent was added, the TG curve also shows significantly decrease. The TG curve stops falling at about 200 °C, and the decrease extent is close to 85%. However, the TG decrease extent is greater than that of the former TG curve. The main reason for this difference is the addition of DMSO solvent in the precursor solution. For the DMSO and DMF mixed solvents, the solvents volatilization temperature was increased to the high temperature zone about 200 °C when compared with the single DMF solvent. Here, the boiling point of DMSO solvent is 189 °C, which is higher than the DMF solvent. The mixed solvents evaporation temperature increased, and the weight loss extent also increased. There are also two endothermic peaks in the DSC curve (see in Figure 3b). The endothermic peaks centers are moved to the higher temperature region when compared with the DSC curve with single DMF solvent.

When the temperature increases, there is no obvious change in the two TG curves in a very short range of temperature. When the temperature increased from 320 °C to 350 °C, there is a slight decrease in the TG curve (see in Figure 3a). In Figure 3b, the TG curve just shows a very weak decrease, and it is almost invisible. This process is corresponding to the decomposition of CH_3_NH_3_PbI_3_. The organic amine salt was decomposed into organic amine and HI gas, which were volatilized. When the temperature increased from 460 °C to 560 °C, there is an obvious decrease in the two TG curves. Corresponding to the DSC curves, there is an endothermic behavior. This process corresponds to the volatilization of I in PbI_2_. Compared with the TG-DSC patterns of the solutions before and after the DMSO solvent added, it could be clearly seen that the evaporation rate of the solvent was slowed down, which contributed to the crystal nucleation and growth.

### 3.2. X-ray Diffraction Analysis

**Figure 4:**
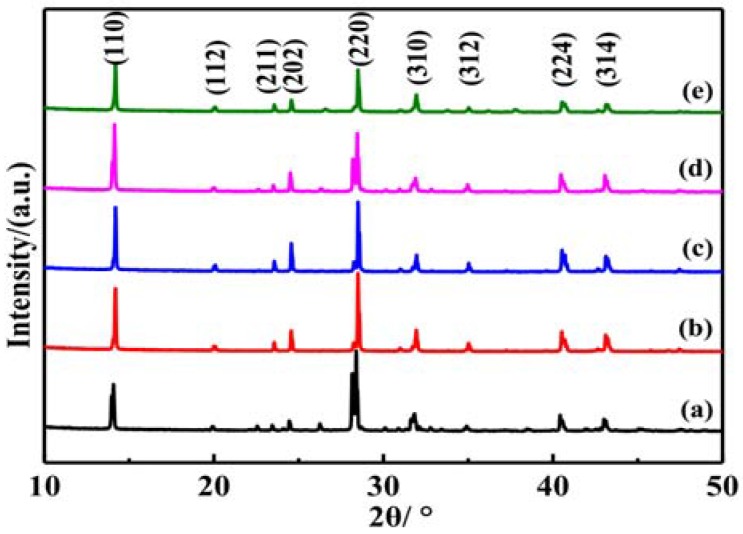
The XRD pattern of perovskite thin films with different ratios of DMSO (**a**) 0%; (**b**) 5%; (**c**) 10%; (**d**) 15%; (**e**) 20%.

Figure 4 shows the X-ray diffraction (XRD) patterns of the perovskite films with different ratios of DMSO solvent added. Ratios of DMSO solvent added were set as 0%, 5%, 10%, 15%, and 20%, respectively. It can be seen that there are the diffraction peaks around 2θ with 14.20°, 20.08°, 23.51°, 24.55°, 28.58°, 28.58°, 35.1°, 40.8°, and 43.0°, which can be assigned to the (110), (112), (211), (202), (220), (310), (312), (224), and (314) planes of the tetragonal perovskite structure [33,34]. The narrow and sharp diffraction peaks showed high crystallinity and orientation. In the Figure 4, the (110) and (220) diffraction peaks intensity is higher than the other peaks. So, in the following discussion, we mainly analyzed the variation of (110) and (220) diffraction peaks. When the ratios of DMSO solvent increased from 0% to 20%, the (110) and (220) diffraction peaks intensities show firstly an increase and then decrease. It was indicated that with the ratio of DMSO solvent added increases, the perovskite film growth along the direction vertical to the substrate was firstly gradually enhanced and then declined. These results confirmed that the ratios of DMSO solvent added have an important regulation effect on the crystallinity of CH_3_NH_3_PbI_3_ film. It was also reported that there are some differences between the cuboid and nanowire perovskite nanocrystals in the XRD patterns [19]. The strongest peak (220) in cuboid particle film becomes weaker in the nanowire film and the (310) peak becomes stronger.

To further investigate the effect of DMSO solvent added on the perovskite film crystal structure, we calculated the lattice strain and analyzed the lattice strain evolution. XRD peak profile analysis is a simple and powerful method to evaluate the peak broadening with crystallite size and lattice strain due to dislocation [35]. Lattice strain is a measure of the distribution of lattice constants arising from crystal imperfections, such as lattice dislocation. The peak broadening of the XRD pattern with different ratios of DMSO solvent added can be used to estimate the lattice strain and crystallite size of the perovskite thin films. During the perovskite thin film growth, there is internal stress in the film. According to Williamson and Hall, the diffraction line broadening is due to crystallite size and strain contribution [35]. The average crystallite size can be obtained from the Debye-Scherrer Equation (1), and the lattice strain was calculated by the following Williamson-Hall Equation (2) [31,35]:

(1) $$D=\frac{0.89\lambda }{\beta \cos\theta }$$

(2) $$\frac{\beta \cos\theta }{\lambda }=\frac{1}{D}+\frac{\varepsilon \sin\theta }{\lambda }$$

Here, D is the crystallite size. *β* is the full width at half maximum (radian) of the diffraction peak. *λ* is the wavelength of the X ray. *θ* is the half-diffraction angle of the crystal plane and *ε* is the lattice strain. The *β* value equals the sample half maximum (radian) of the diffraction peak subtracts the width of the X ray source of the instrument itself. The average crystallite sizes D of the different ratios of DMSO solvent added were 60 nm, 75 nm, 81 nm, 112 nm, and 135 nm, respectively, which were calculated on the basis of the above Equation (1). The average crystallite size gradually increases as the ratios of DMSO solvent being added increases. In the perovskite thin films, the large crystallite sizes can reduce the grain boundaries number, thus restrains the carriers recombination, and increases the carrier mobility [36].

**Figure 5:**
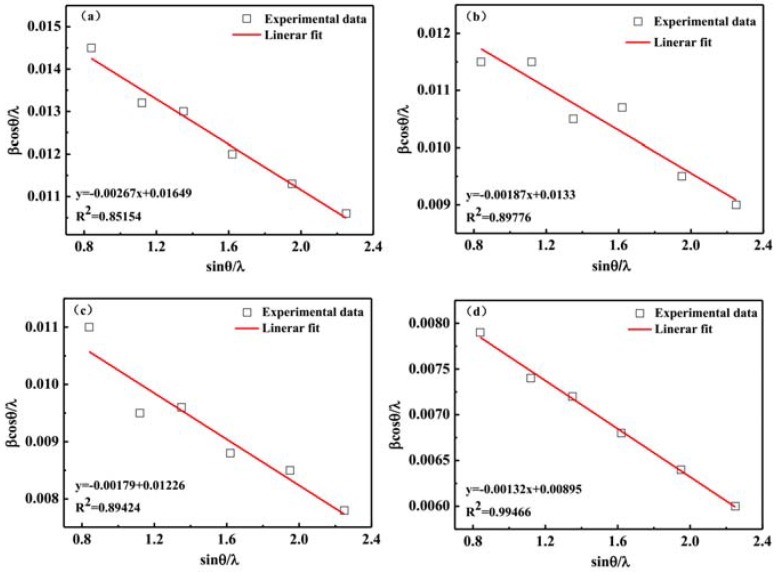

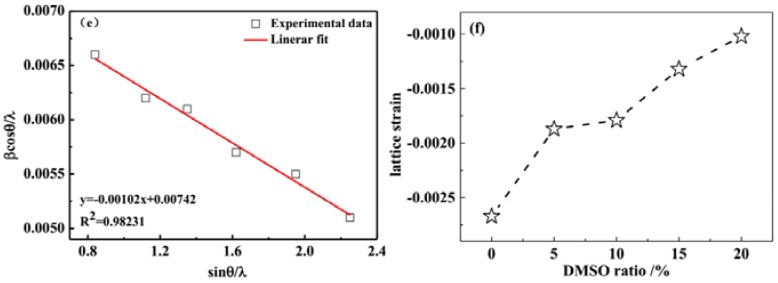
The lattice strain analysis plots for different ratios of DMSO added. (**a**) 0%; (**b**) 5%; (**c**) 10%; (**d**) 15%; (**e**) 20%. (**f**) The relationship between the lattice strains and DMSO ratios.

The slope of the graph (sin*θ*/*λ* versus *β*cos*θ*/*λ*) is presented in Figure 5. The slope of the line corresponds to the lattice strain, and the reciprocal of the intersection value of the line and the y-axis corresponds to the average crystallite size. A straight line was fitted to the data points for the estimation of the lattice strain. The slope of the line is negative. It shows that the residual lattice strain for the perovskite films is compressive in nature. Based on this Williamson-Hall equation, the lattice strain of ZnO nanoparticles also has been reported [35]. During the perovskite thin film annealing progress, the residual lattice strain also is compressive [31]. In Figure 5f, the lattice strains increased with the ratios of DMSO solvent added. The crystallite size also increased with the ratios of DMSO solvent added.

### 3.3. Scanning Electron Microscopy Analysis

**Figure 6:**
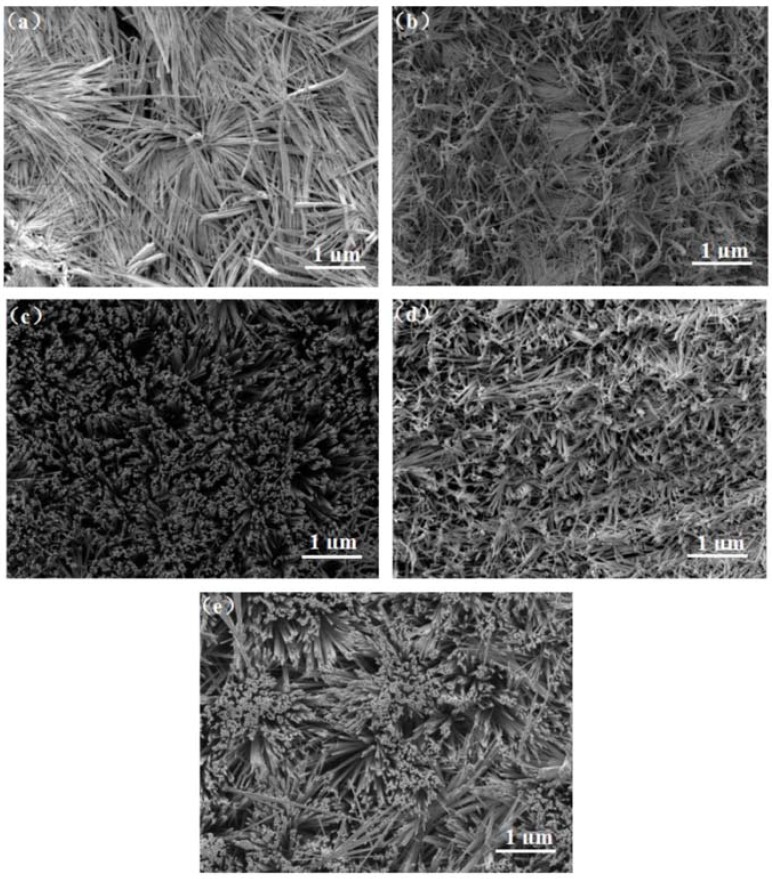
The scanning electron microscope (SEM) images of perovskite thin films with different ratios of DMSO (**a**) 0%; (**b**) 5%; (**c**) 10%; (**d**) 15%; (**e**) 20%.

Figure 6 shows the SEM images of the CH_3_NH_3_PbI_3_ nanowires thin film. The DMSO solvent has a significant impact on the morphology of the perovskite thin films. The uniformity and orderliness of the perovskite nanowires increase first and then decrease with the ratios of DMSO solvent added. When the ratio of the DMSO solvent added is 0%, the diameter of the perovskite nanowires uneven and most of them are laid on the substrates (see in Figure 6a). The substrate was mostly fully covered by the scattered nanowires. When the ratio of DMSO solvent is 5%, the nanowires with curved shape covered the entire substrate surface, and some of the nanowires are vertical to the substrate. Some of the nanowires diameters are smaller than that in Figure 6a. When the ratio of DMSO solvent being added increased to 10% (see in Figure 6c), we can see the crystallinity and uniformity of the perovskite nanowires are significantly improved. Most of the nanowires are perpendicular to the substrate and form nanowire forest. The amount of DMSO introduced in the system is very critical. In this work, the best ratio of DMSO solvent added is 10%. The perovskite nanowires grow vertical to the substrate forming a perfect forest structure. The solvent DMSO addition will slow down the evaporation of the solvent and contribute to the nucleation and grain growth [37]. The perovskite thin film with nanowire forest is favorable for the carriers fast transport. When the ratio of DMSO solvent being added further increased to 15% (see in Figure 6d), the orientation of the perovskite nanowire film is lowered, but it is still substantially vertical to the substrate growth. Some nanowires top ends are clustered together. When the DMSO ratio increased to 20% (see in Figure 6e), the morphology of the perovskite film changes, and the uniformity of the film is lowered due to clustering of the nanowires. The DMSO solvent played an important role in regulating the ordered structure of the perovskite nanowires growth. When comparing the morphology of the above nanowires perovskite film, when the ratio of DMSO solvent added is 10%, the perovskite thin film has the best orientation and uniformity, formed nanowires forest.

The crystallization process of the nanowires perovskite thin film can be adjusted by changing the ratios of DMSO. The intermediate phase (CH_3_NH_3_I·PbI_2_·DMSO) is introduced to cause the originally disordered crystals been oriented to achieve uniform coverage of the substrate. Therefore, an appropriate ratio of organic solvent DMSO is beneficial to regulate crystal growth and obtained the high-quality ordered nanowires structure of perovskite film, the nanowire structure vertical to the substrate is favorable for the rapid separation of the holes and electrons, increases the coverage of the film surface and the intensity of light absorption. Perovskite nanowires thin films are used as the light absorption layer in the device. Optical absorption is a key parameter to characterize its performance. Therefore, we have tested and analyzed the optical absorption properties of the thin film.

### 3.4. Optical Performannce Analysis

**Figure 7:**
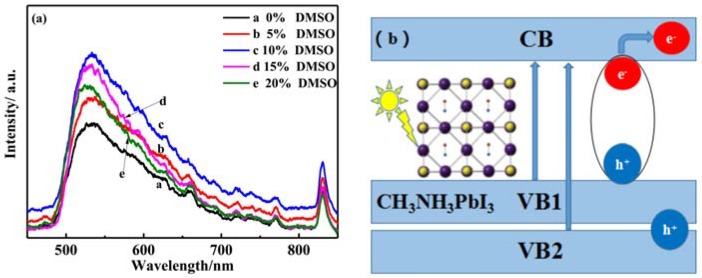
(**a**) Fluorescence emission spectra of perovskite solution precursor (the excitation wavelength was 325 nm); (**b**) The band distribution of the perovskite.

The perovskite thin films in the solar cell are mainly used to absorb light and generate carriers in the perovskite solar cells. So, the optical performance also has been carried out. Firstly, optical properties of perovskite precursor solution were characterized. Figure 7a shows the fluorescence spectra of perovskite precursor solution with different ratios of DMSO solvent. In the visible range of the spectrum, the fluorescence intensity of the precursor solution first increases and then declines with increasing of the ratios of DMSO solvent. When the ratio of DMSO was 10%, the fluorescence intensity was the strongest. It was reported that the photoluminescence (PL) intensity of the perovskite film enhances, indicating that the perovskite film quality is becoming better [38]. The defect density is thus reduced to suppress non-radioactive recombination in the absorber layer [39]. The non-radiative composite channel was suppressed. The PL intensity is related to the carrier lifetime, and the stronger PL intensity corresponds to the longer carrier lifetime [11]. When the PL intensity increased, the defect density reduced and the carrier lifetime prolonged. Less carrier recombination was beneficial to the charge transfer characteristics. The PL intensity of the perovskite sample decrease, revealing many defects may occur in the bulk or interface of the perovskite thin film [38]. Khadka et al. [40] have been reported the effect of carrier transport on the performance of the perovskite device, with the polymer polytriarylamine (PTAA) as the hole transport layer in the perovskite device performance, the interface quality and efficient carrier transport can be improved.

There are two emission peaks that are centered at 530 nm and 830 nm in Figure 7a, which corresponding to the carriers transition of the valence band VB2 and VB1 to CB1 [41]. Figure 7b is the energy band schematic diagram of the perovskite CH_3_NH_3_PbI_3_. It was also reported that there are two distinct peaks located at 480 nm and 760 nm in the ultraviolet to near infrared absorption spectrum for the CH_3_NH_3_PbI_3_ thin films [41]. In the perovskite thin film, the luminescence process is owing to the carriers bimolecular recombination [31]. There are two valence bands VB1 and VB2 in the Figure 7b. The lower valance band VB2 position is −6.5 eV and the higher valance band VB1 position is −5.6 eV. The conduction band position is −3.9 eV [41]. When emission light wavelength is 530 nm, it is corresponding to the carriers transition from conduction band C1 to valence band B2. When emission light wavelength is 830 nm, it is corresponding to the carriers transition from conduction band C1 to valence band B1. The band gap of perovskite film can be obtained by calculated the formula: λ = 1240/Eg. When the emission light wavelength is 830 nm, the band gap of perovskite CH_3_NH_3_PbI_3_ is 1.49 eV. This calculated band gap width is approaching the perovskite band gap value of 1.55 eV. The fluorescence effect of the perovskite precursor solution needs further study and confirm. The PL spectroscopy is also affected by the excitation wavelength and the structure and properties of the perovskite thin films. When the excitation wavelength is 532 nm, the PL peaks shift to short wavelength (about from 770 nm to 720 nm) gradually when decreasing the 2D perovskite nanosheets thickness [16]. The PL emission peaks changed with the different halogen elements in the perovskite. The peaks of PL emission from CH_3_NH_3_PbBr_3_, CH_3_NH_3_PbBr_x_I_3−x_, and CH_3_NH_3_PbI_3_ 2D nanosheets appeared at approximately 535 nm, 660 nm, and 730 nm, respectively [16].

**Figure 8:**
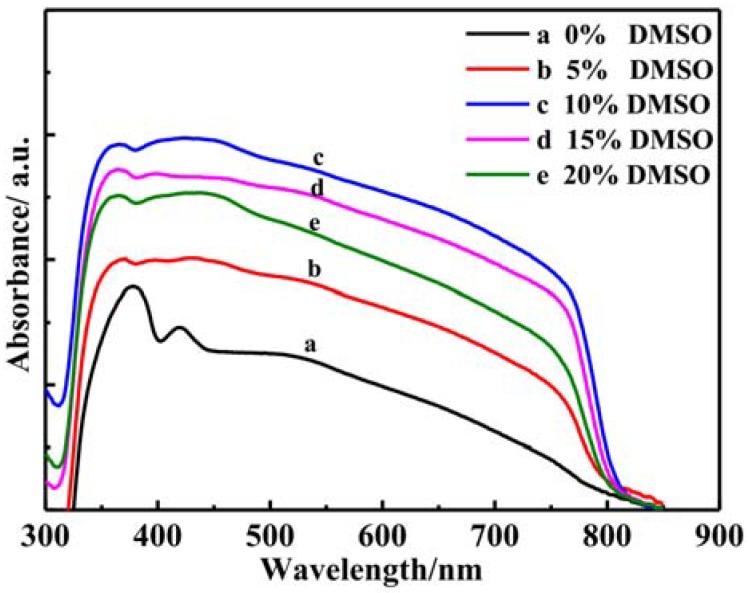
UV-vis absorption spectra of perovskite nanowires film with different ratios of DMSO added.

As the light absorption layer, perovskite thin film is mainly due to their excellent photoelectric properties. Here, the absorption properties of the nanowires perovskite thin films were studied. Figure 8 shows the light absorption spectrum of the perovskite nanowires thin films with different ratios of DMSO solvent added. The light absorption range covers the entire visible light. With the ratios of DMSO solvent added, the light absorption intensities present increases first and then decrease. When the ratio of DMSO solvent added is 10%, the perovskite nanowires thin film shows the strongest light absorption intensity. The light absorption performance is one of the most important indicators to characterize the photoelectric properties of the perovskite thin films. The optical properties of the perovskite thin films are closely related to the surface morphology. When combined with the perovskite nanowires morphology, it can be confirmed that perovskite thin film with nanowires forest showed the best optical absorption performance.

The ordered nanowires structures of the perovskite thin films are closely related to the preparation process. During the crystallite grow up, proper ratio of DMSO solvent added can slow down the solvent evaporation and promote the crystallinity. The ordered and dispersed nanowires forest has the strong light absorption. This is like a “light trap” effect, which can significantly improve the light absorption capacity. The ordered and dispersed nanowires forest perovskite thin film exhibits strong light absorption properties, which can reduce the thickness of the film and promote the carrier collection. Perovskite nanowires forest with less than 1 micron can absorb the entire visible spectrum. To sum up, when the ratio of DMSO solvent added is 10%, the light absorption intensity of ordered and dispersed nanowires forest perovskite thin film reached a maximum value. This perovskite thin film with ordered and dispersed nanowires is suitable as the light absorption layer in the solar cell.

### 3.5. The Transient Photocurrent and J-V Analysis

**Figure 9:**
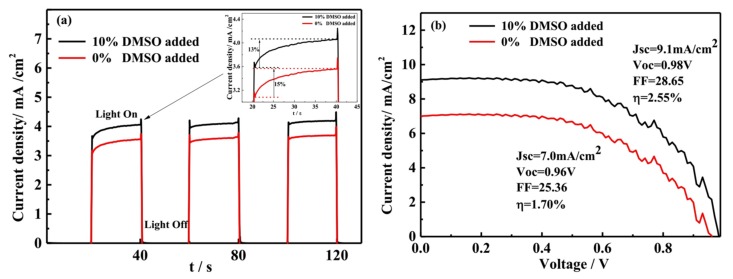
(**a**) The transient photocurrent-time; (**b**) The J-V curves of the perovskite solar cell.

As we know, the perovskite thin film not only can transport electrons, but also transport holes. Based on this viewpoint, we fabricated the simple structure nanowires perovskite thin film solar cells. The device structure is FTO/perovskite/Ag, and the device effective area is 0.25 cm^2^. To further investigate the electrical properties of the ordered and dispersed nanowires forest perovskite thin film, the photocurrent as a function of time and the J-V characteristics while using simulated sunlight at AM1.5G (100 mW/cm^2^) were measured, as shown in Figure 9. Figure 9a shows the transient current density as function of time, and the constant voltage applied across on the device is 0.5 V. The black line represents the perovskite thin film solar cell with 10% ratio of DMSO solvent added. The red line represents the device without DMSO solvent added. In the absence of light environment, the transient photocurrent of the devices is zero, no matter whether the DMSO added. Without light, so there is no photocurrent generation. The photoelectric effect inside the device was excited by the irradiation of the standard simulated sunlight. Turn on the light, there will be photocurrent current appearing. The transient photocurrent density is different for these two devices, mainly due to the difference in the morphology of the optical absorption layers. For the three transient photocurrent cycle tests, the device with 10% ratio of DMSO added has the higher current density. That is to say, the perovskite thin film with ordered and dispersed nanowires has stronger photogenerated carriers generation and collection ability. It is very suitable to use as the light absorption layer in the solar cell. In the first transient photocurrent measurement, the current density is not invariable, but it has a slight increase tendency (see illustration in Figure 9a). The device with 10% ratio of DMSO solvent added, the transient current density is increased by 13%. The other device with no DMSO solvent added, the transient current density is increased by 15%. However, for the second and third time light on during the transient photocurrent measurement, the current density values did not show obvious change, mostly reached a stable value for the two devices. The possible reason for this phenomenon is that the solar cell maybe failed to reach the steady state during the initial test. The transient current density is not only related to the perovskite film properties, but also to the light intensity. It has been reported that the current increased with the light intensity or input power increase [16].

For the perovskite solar cells, the J-V measurements also have been carried out. Figure 9b shows the J-V curves of the two devices. When the ratio of DMSO is 10%, the device power conversion efficiency is 2.55%. When the ratio of DMSO is 0%, the device power conversation efficiency is 1.70%. When the DMSO solvent was added 10% ratio in the mixed solvents, the device power conversation efficiency is increased by 50%. For the two I-V curves, the main difference is the photocurrent value, and the voltage difference is very small. The difference in photocurrent is due primarily to the difference in optical absorption properties. The perovskite thin film with a 10% ratio of DMSO showed stronger optical absorption, stronger photogenerated carrier generation, and collection ability. The ordered and dispersed nanowires perovskite thin film is more suitable to use as the light absorbing layer in the solar cell. The power conversation efficiency of the two devices is not very high. It is related to the devices preparation progress and the devices structure. Perovskite materials and devices are prepared in the atmospheric environment, and there is also no electron transport layer and hole transport layer in the devices. The perovskite solar cell performance can be improved when added to the transport layer and hole transport layer. The power conversion efficiency of the nanowires perovskite thin film solar cell needs to be improved further by further optimizing the preparation progress.

## 4. Conclusions

In summary, perovskite thin films consisting with 1D nanowires and simplified structure solar cells without electron transport layer (ETL) and hole transport layer (HTL) have been prepared with solution method under an atmospheric environment. The effect of DMSO solvent added ratios on the structure, morphology, and optical properties of perovskite thin films has been investigated. The fluorescence intensity of the precursor solution shows firstly increases and then declines with the ratios of DMSO solvent increase under 325 nm excitation. The optical absorption intensity of the perovskite thin films with 1D nanowires shows an increase first and then decrease with the DMSO ratios increase. Appropriate ratio of DMSO solvent added is beneficial to growth the perovskite thin films with ordered and dispersed nanowires forest. When the ratios of DMSO solvent added increased, the uniformity and orderliness of the perovskite nanowires present first increase and then decrease. The ratio of DMSO solvent that is added is 10%; the fluorescence emission intensity and the optical absorption intensity reached the maximum. At the same time, the perovskite nanowires have the best order and dispersibility. The perovskite thin film with ordered and dispersibility nanowires have the strongest optical absorption ability when compared with the disordered nanowires perovskite thin films. The ordered and dispersed nanowires forest plays an optical trapping role. For the simplified structural device with ordered nanowires perovskite thin film as absorption layer, the power conversation efficiency is 2.55%. This power conversation efficiency is increased by 50% when compared with the device with disordered nanowires perovskite thin film. The ordered and dispersed nanowires forest structure played a key role in improving the device performance.
